# Bioelectrical Recovery After Knee Injury in an Elite Alpine Skier: A Longitudinal 19-Frequency Bioelectrical Impedance Vector Analysis Case Report

**DOI:** 10.3390/jcm15187231

**Published:** 2026-09-17

**Authors:** Stanislav Štuhec, Tina Robnik, Mateusz Jopek, Krzysztof Mackala

**Affiliations:** 1 Faculty of Sport, University of Ljubljana, Gortanova 22, 1000 Ljubljana, Slovenia; stanislav.stuhec@fsp.uni-lj.si; 2 Department of Physiotherapy, Alma Mater Europaea University, Slovenska Street 17, 2000 Maribor, Slovenia; tina.robnik@gmail.com; 3 Department of Individual and Team Physical Activities, Wroclaw University of Health and Sport Sciences, al. Ignacego Jana Paderewskiego 35, 51-612 Wrocław, Poland; mateusz.jopek@awf.wroc.pl

**Keywords:** bioelectrical impedance, BIVA, A19FBIVA, phase angle, reactance, return-to-play, rehabilitation, knee injury, alpine skiing, limb symmetry index, case report

## Abstract

Return-to-play (RTP) decisions after severe knee injury in elite athletes remain challenging because functional recovery may not coincide with physiological tissue recovery. This longitudinal case report evaluated Advanced 19-Frequency Bioelectrical Impedance Vector Analysis (A19FBIVA) as a non-invasive sensor-based approach for monitoring segmental lower-limb bioelectrical changes in an elite female alpine skier following a complex right knee injury. Segmental resistance (R), reactance (Xc), phase angle (PhA), and inter-limb asymmetry were assessed with a Seca mBCA 515 analyzer at 19 frequencies (1–1000 kHz) at four time points: 5 days pre-injury, 26 days post-injury, 64 days post-injury, and 135 days post-injury. The rare pre-injury baseline enabled individualized comparison beyond contralateral symmetry. At the final late-stage rehabilitation follow-up, R in the injured lower-limb segment had normalized or improved, whereas Xc and PhA remained suppressed, indicating an observational bioelectrical pattern consistent with persistent capacitance-related impairment rather than direct evidence of localized knee-tissue healing. The uninjured limb also deteriorated during early rehabilitation, demonstrating that contralateral comparison alone may underestimate residual deficits. These findings support the exploratory concept of a ‘reactance lag’ and indicate that A19FBIVA may provide a useful complementary monitoring layer, alongside clinical examination, imaging, functional testing, and sport-specific assessment. Larger prospective studies are required to validate thresholds, reliability, and prognostic value.

## 1. Introduction

Return-to-play (RTP) decision-making after knee injury remains one of the most complex challenges in elite sports medicine. Functional milestones such as hop performance, isokinetic strength, and limb symmetry indices are frequently used to support clearance, but these criteria may not fully reflect all physiological or tissue-related processes occurring during recovery [1,2,3,4,5,6,7,8,9,10,11,12]. Athletes can develop compensatory movement strategies or benefit from bilateral detraining, thereby achieving apparent functional symmetry despite residual deficits in tissue quality, cellular integrity, or local fluid regulation [13,14,15,16,17,18].

This problem is especially relevant in alpine skiing. Ski racing exposes the knee to high external loads, eccentric muscle actions, torsional stress, vibration, and multiplanar forces [19,20,21,22,23,24,25,26]. A limb that appears clinically stable or functionally symmetrical in laboratory testing may still be exposed to mechanical demands during high-speed turns, edging, landing, and changing snow conditions. Consequently, RTP decisions in elite skiing require both functional and physiological information.

Bioelectrical impedance analysis (BIA) is a non-invasive method that measures the electrical characteristics of biological tissues. Resistance (R) represents the opposition to electrical current and is mainly affected by fluids and electrolytes that conduct electricity. Reactance (Xc) indicates the capacitive qualities related to cell membranes and tissue interfaces. The phase angle (PhA), calculated from R and Xc, is often used as a combined indicator of cellular health and membrane integrity [27,28,29,30,31,32,33,34,35,36,37,38,39,40]. In sports rehabilitation, these measurements can offer indirect insights into conditions like edema, tissue conductivity, muscle changes, and tissue capacitance [28,29,41,42,43,44].

Advanced 19-Frequency Bioelectrical Impedance Vector Analysis (A19FBIVA) is especially useful in rehabilitation monitoring because it measures impedance over a wide frequency range. Lower frequencies are more responsive to extracellular pathways, while higher frequencies reveal additional insights into the total conductive tissue and intracellular spaces [27,28,29,30,31,36,37,41,45,46,47]. This multifrequency method can distinguish fluid changes from capacitance-related electrical patterns, but it should be used for segmental lower-limb assessment rather than examining a specific knee structure.

Sensor-based rehabilitation monitoring is gradually expanding beyond laboratory testing. Wearable inertial measurement units (IMUs), for instance, have been used to measure lower-limb joint angles during landing and cutting tasks, which are important for injury-risk assessment and return-to-play decisions [48]. While these systems offer movement-related data during dynamic activities, A19FBIVA focuses on resting segmental bioelectrical information. Therefore, these approaches should be seen as complementary parts of a comprehensive sensor-based rehabilitation framework rather than rivals.

This longitudinal case report aimed to assess whether A19FBIVA could detect segmental bioelectrical changes in the lower limb during an elite alpine skier’s post-injury rehabilitation following a complex knee injury. We hypothesized that Xc and PhA would exhibit ongoing bioelectrical differences in the late stages of rehab, though we did not believe these changes directly indicated knee-tissue healing or separate RTP readiness.

## 2. Materials and Methods

### 2.1. Study Design and Reporting Standard

This study was conducted as a longitudinal case report on a single athlete, offering insights into clinical rehabilitation monitoring. It adhered to CARE reporting standards where relevant [49]. Segmental bioelectrical impedance measurements were taken before and after a serious knee injury, enabling comparison of the injured limb with the uninjured side and the athlete’s pre-injury baseline. Since the Seca mBCA 515 measures impedance at the segmental lower limb level rather than the knee joint specifically, all results are understood as changes in the bioelectrical properties of the lower limb segment following injury, rather than direct indicators of local knee tissue healing.

### 2.2. Participant and Injury History

The participant was a female elite alpine skier competing at the Slovenian national level. She sustained a complex right knee injury, as documented in the available records, involving a tibial fracture with associated strain of the knee ligaments, tendons, and muscles. The injury required approximately one month of rest and immobilization with a non-walking knee brace, followed by medically supervised, progressive rehabilitation. The available dataset did not include a more detailed fracture classification, imaging series, or complete documentation of whether all management components were surgical or conservative; this limitation is now explicitly acknowledged. The participant is also a co-author of this case report and provided written informed consent for the use of her anonymized demographic, health, and measurement data for scientific publication. Clinical and measurement data are presented only to the extent necessary for the scientific interpretation of the case.

Because the participant was also a co-author, we addressed potential reporting bias by focusing the analysis on objective, device-derived bioelectrical variables rather than subjective recovery reports. Co-authors who were not the participant interpreted the data and prepared and reviewed the manuscript, and the manuscript transparently discloses the participant’s dual role. Participant and injury characteristics are summarized in Table 1.

### 2.3. Bioelectrical Impedance Device

Bioelectrical impedance was assessed using the Seca mBCA 515 multifrequency analyzer (Seca GmbH, Hamburg, Germany). The device uses an eight-electrode configuration and enables segmental analysis of the left leg, right leg, left arm, right arm, trunk, left body side, right body side, and whole body. Measurements were performed across 19 frequencies: 1, 1.5, 2, 3, 5, 7.5, 10, 15, 20, 30, 50, 75, 100, 150, 200, 300, 500, 750, and 1000 kHz [30,31,36,37,41,45,46,47].

### 2.4. Measurement Conditions

To minimize biological variability, we performed measurements under standardized conditions. The athlete was measured standing, barefoot on the platform electrodes, with the hands gripping the hand electrodes. Measurements were performed at similar times of day, in a resting state, with no intense exercise in the preceding 12 h, normal hydration, and bladder voiding before testing [30,31,36,37]. At each measurement, the athlete was instructed to stand as symmetrically as possible, with both feet fully contacting the platform electrodes and both hands maintaining stable contact with the hand electrodes. Measurements were performed only when the athlete could maintain the standing position safely and without external support. Stable posture and adequate electrode contact were verified visually by the examiner. Integrated load-sensor feedback or quantitative weight-distribution data were not available.

Food and fluid intake, body-mass changes, medication use, environmental conditions, detailed recent training load, and menstrual-cycle phase were not fully standardized or recorded at each time point. We recognize these factors as potential sources of longitudinal measurement variability and note them as limitations. The A19FBIVA measurement protocol is summarized in Table 2.

### 2.5. Measurement Timeline

**Figure 1:**
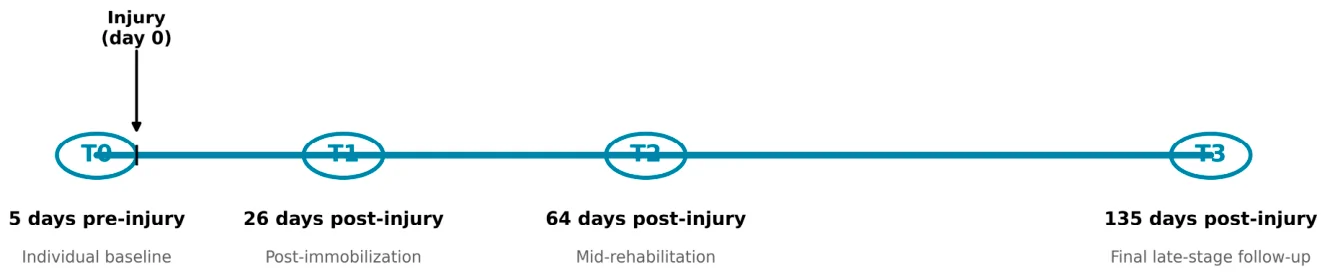
Longitudinal measurement timeline from pre-injury baseline to the final late-stage rehabilitation follow-up. T0—baseline; T1—post-immobilization/early rehabilitation; T2—mid-rehabilitation; T3—final late-stage rehabilitation follow-up.

Four measurements were analyzed: T0, 5 days pre-injury; T1, 26 days post-injury; T2, 64 days post-injury; and T3, 135 days post-injury. The pre-injury measurement provided a rare individualized baseline for interpreting longitudinal bioelectrical recovery. Because no concurrent standardized functional testing, strength assessment, imaging, or formal RTP clearance battery was available at T3, this time point is described as the final late-stage rehabilitation follow-up rather than confirmed RTP readiness. The longitudinal measurement timeline is presented in Figure 1.

### 2.6. Variables and Data Processing

Raw R and Xc values were extracted for the injured right lower-limb segment and the uninjured left lower-limb segment at all 19 frequencies. PhA was calculated from R and Xc using the formula: PhA (degrees) = arctangent(Xc/R) × 180/π. Percentage change from the pre-injury baseline was calculated as: [(post-injury value − b-aseline value)/baseline value] × 100. Inter-limb asymmetry was calculated as: [(injured limb − uninjured limb)/uninjured limb] × 100. We focused on 50 kHz because it is widely used in BIA research, while retaining additional frequency-dependent patterns to characterize the A19FBIVA profile [27,28,29,30,31,32,33,34,36,37].

### 2.7. Data Analysis

Because the objective was to characterize within-athlete signal evolution, the analysis prioritized transparency and reproducibility over statistical complexity. For each time point, raw segmental R and Xc values were inspected for the injured right leg and uninjured left leg. Percentage changes were then interpreted relative to the pre-injury value, which served as the individualized reference condition. When possible, we retained both absolute values and percentage changes because the direction of change may have different physiological meanings depending on the variable.

Inter-limb asymmetry was used as a secondary descriptor, not as the sole indicator of recovery. This distinction was important because the contralateral limb was expected to be affected by reduced training exposure and systemic deconditioning. Accordingly, we interpreted a reduction in side-to-side asymmetry as meaningful only when accompanied by improvement relative to the athlete’s baseline.

Given the single-athlete design, we did not apply inferential statistics. Results are presented descriptively as raw values, percentage changes from baseline, and inter-limb asymmetry. The primary analytical focus was the dissociation between R and Xc/PhA trajectories. Each time point represented one measurement session; repeated sweeps sufficient to calculate within-session technical error, coefficient of variation, standard error of measurement, or minimal detectable change were not available. Therefore, we interpret small longitudinal differences cautiously, and we present the results as hypothesis-generating observations rather than definitive physiological effects.

## 3. Results

### 3.1. Resistance and Reactance Dynamics

**Figure 2:**
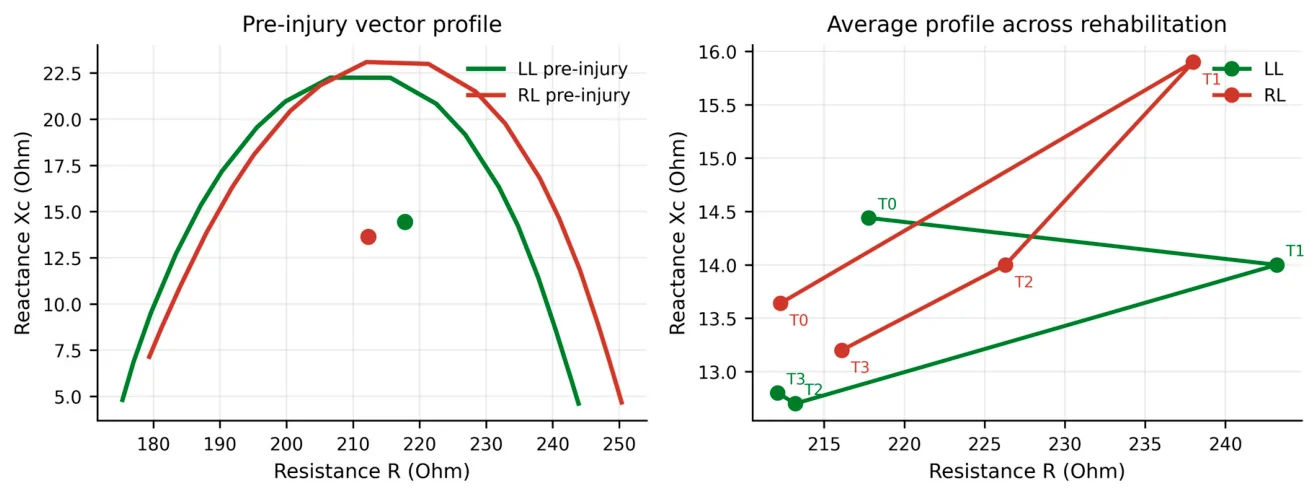
Bioelectrical impedance vector profile of the injured and uninjured lower limbs across 19 frequencies. The (**Left**) panel presents the pre-injury relationship between resistance and reactance, whereas the (**Right**) panel illustrates longitudinal changes across the rehabilitation process. RL—right leg; LL—left leg; R—resistance; Xc—reactance.

**Figure 3:**
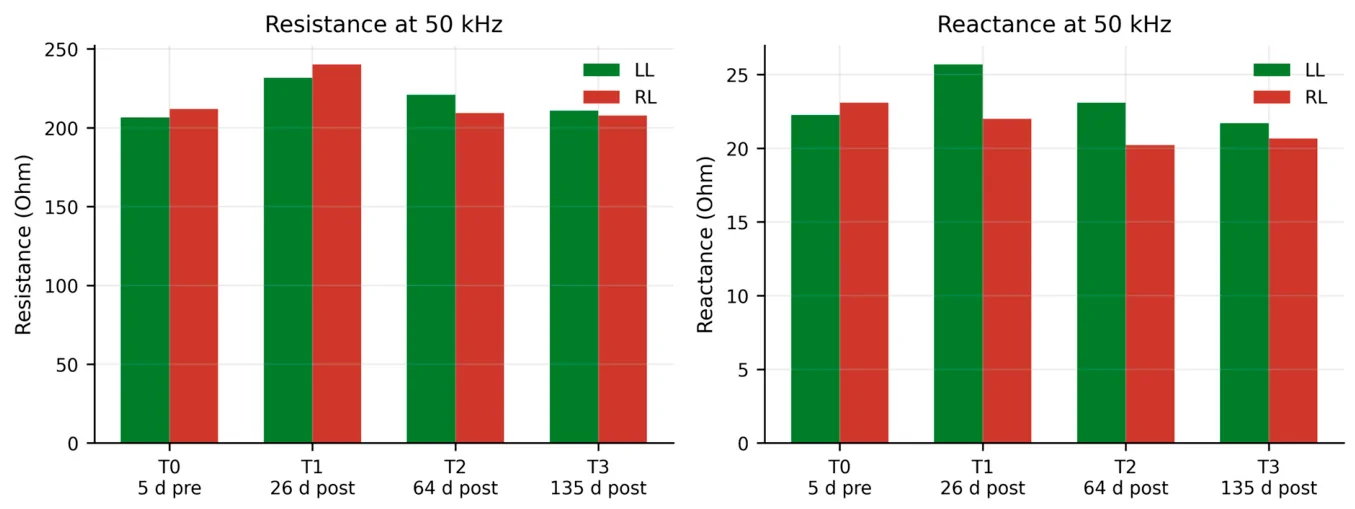
Longitudinal changes in resistance and reactance at 50 kHz in the injured and uninjured lower-limb segments. Resistance values peaked after immobilization and progressively decreased during rehabilitation, whereas reactance remained lower in the injured lower-limb segment, indicating persistent suppression of capacitance-related bioelectrical properties.

**Figure 4:**
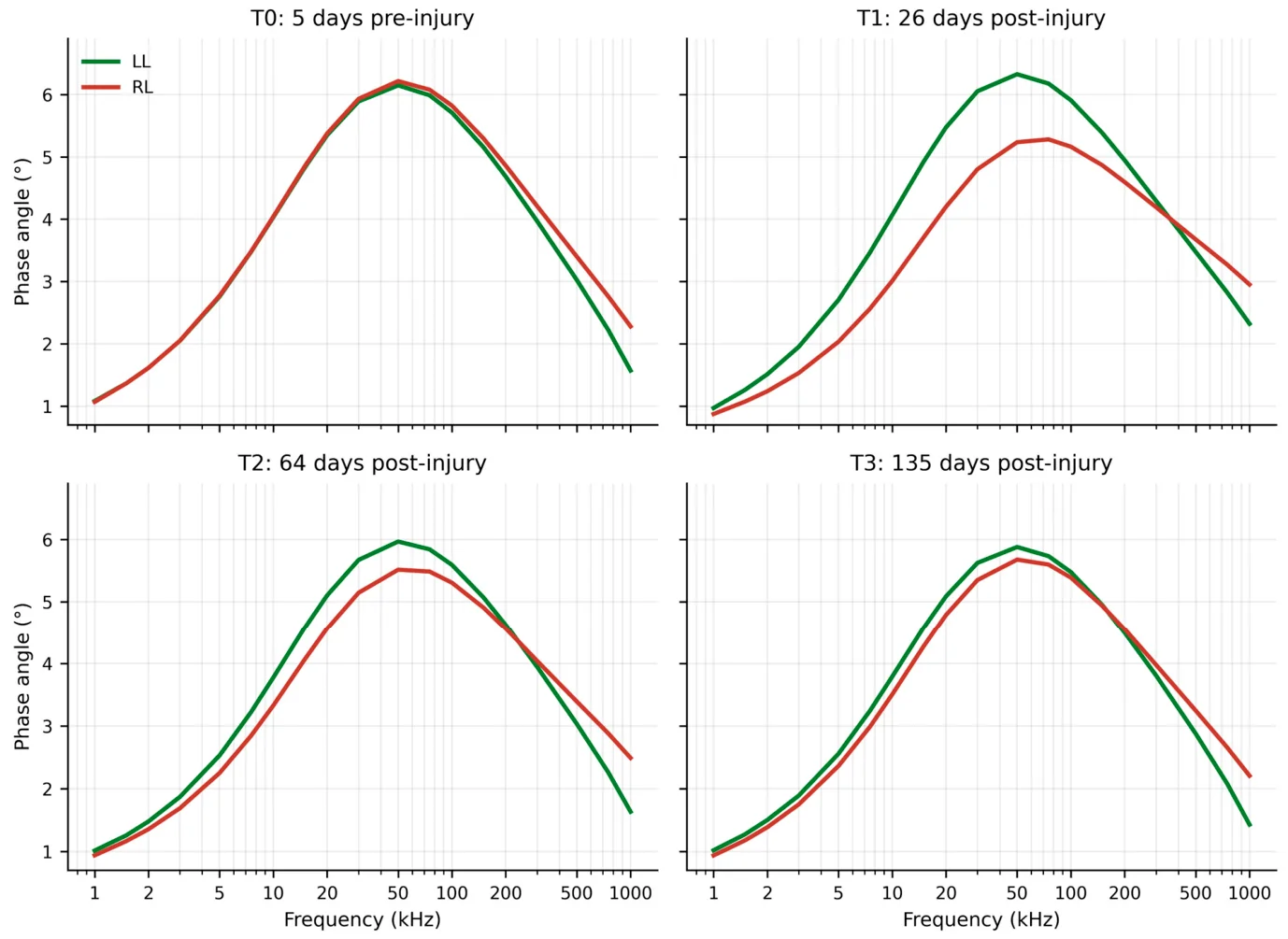
Phase angle profiles of the injured and uninjured lower-limb segments across 19 frequencies at four measurement time points. The greatest inter-limb divergence occurred after immobilization, with partial but incomplete normalization by the final late-stage rehabilitation follow-up. PhA—phase angle.

The injured lower-limb segment showed divergent patterns between R and Xc. At T1, R increased, consistent with altered conductive tissue volume and disuse-related changes after immobilization. In contrast, Xc decreased, indicating reduced capacitance-related bioelectrical properties. By T3, R had normalized or improved relative to baseline, whereas Xc remained substantially suppressed. This dissociation suggests that fluid- or volume-related segmental changes may normalize before capacitance-related bioelectrical properties [27,28,29,34,41]. The corresponding longitudinal bioelectrical impedance vector profiles are presented in Figure 2. Longitudinal changes in resistance and reactance at 50 kHz are shown in Figure 3. Phase angle profiles across the 19 measured frequencies are presented in Figure 4.

### 3.2. Reactance Lag

**Figure 5:**
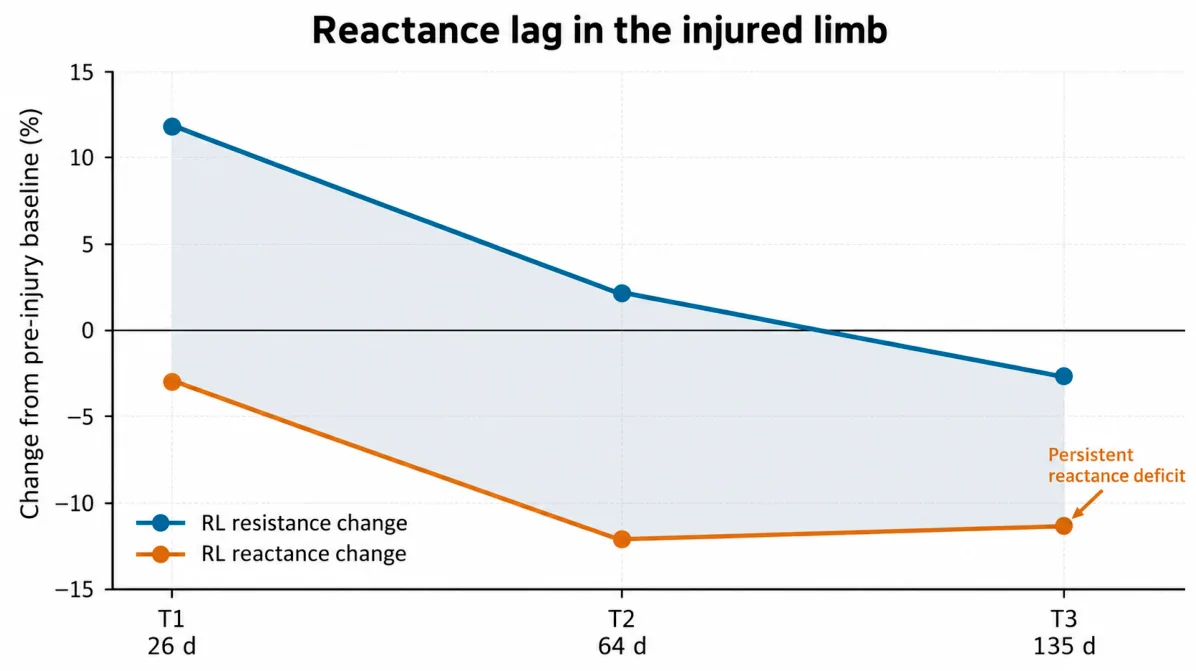
Reactance lag in the injured lower-limb segment. Resistance normalized by the final measurement, whereas reactance remained suppressed, indicating an observational dissociation between conductive and capacitance-related bioelectrical properties.

The main observational finding was the persistence of an Xc deficit despite R normalization. This pattern is described here as a reactance lag, meaning a temporal dissociation between R normalization and persistent Xc suppression in the injured lower-limb segment. At T3, R in the injured lower-limb segment changed by −2.61% relative to baseline, while Xc remained reduced by −11.36%. This temporal dissociation between resistance and reactance is illustrated in Figure 5.

The term ‘reactance lag’ is used descriptively to describe an observed bioelectrical pattern. It does not imply a new diagnostic category or confirm a specific cellular repair mechanism. Without concurrent MRI, ultrasound, histology, or localized tissue validation, interpret this finding as persistent suppression of capacitance-related segmental bioelectrical properties rather than as direct evidence of delayed knee-tissue healing.

A practical implication is that segmental bioelectrical monitoring may provide information not captured by time-based rehabilitation milestones alone. However, because concurrent functional tests, strength measures, imaging, and patient-reported outcome measures were not collected at all time points, the present data cannot demonstrate that A19FBIVA detects deficits missed by standard RTP criteria. Instead, A19FBIVA should be considered a complementary monitoring tool that may help guide hypotheses about load progression and recovery status. The main longitudinal bioelectrical outcomes at 50 kHz are summarized in Table 3.

### 3.3. Phase Angle Recovery

**Figure 6:**
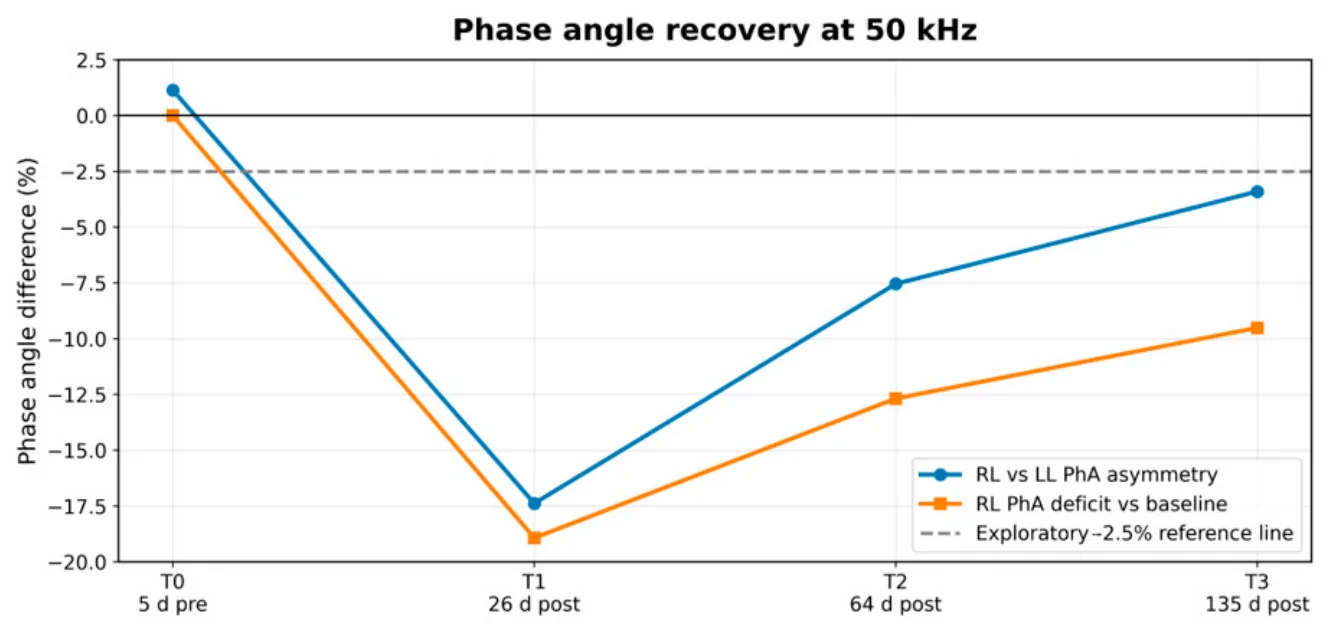
Phase angle deficit and inter-limb asymmetry during rehabilitation. Despite gradual improvement, phase angle remained below pre-injury values at the final measurement. The dashed line is an exploratory −2.5% visual reference and should not be interpreted as a validated clinical threshold.

PhA provided an integrated segmental bioelectrical marker reflecting capacitance-related tissue properties. The injured lower-limb segment exhibited a substantial PhA deficit after immobilization, with gradual improvement during rehabilitation. However, PhA remained below the pre-injury baseline at the final measurement. The injured lower-limb segment showed PhA deficits of −18.93% at T1 and −9.51% at T3 relative to its own baseline, while right-to-left PhA asymmetry was −3.40% at T3. Phase angle outcomes at 50 kHz are summarized in Table 4. The longitudinal phase angle deficit and inter-limb asymmetry are illustrated in Figure 6.

### 3.4. Contralateral Limb Deterioration

The deterioration of the uninjured limb was not treated as a secondary artifact but as an important component of the recovery profile. In a conventional LSI framework, the contralateral limb is frequently assumed to represent the target condition. In the present case, this assumption would be problematic because the uninjured limb also changed during the period of immobilization and rehabilitation.

This finding supports the use of baseline-referenced monitoring whenever possible. If the injured limb is compared only with a deconditioned reference limb, apparent symmetry may be achieved even when both limbs remain below the athlete’s optimal pre-injury status. Therefore, the interpretation of A19FBIVA data should include both side-to-side comparison and within-limb comparison to baseline.

### 3.5. Frequency-Dependent Profile Interpretation

The frequency-dependent figures provide an additional layer of interpretation beyond the 50 kHz summary outcomes. The BIVA-style R-Xc plots show that the relationship between R and Xc shifted across the rehabilitation process rather than changing as a single linear recovery trajectory. This supports the view that the injury and rehabilitation process affected both conductive and capacitive properties of the lower limbs.

The 1 kHz, 50 kHz, and 1000 kHz views serve different interpretive purposes. Additional resistance and reactance profiles at 1 kHz and 1000 kHz are provided in Supplementary Figures S1 and S2, respectively. The 1 kHz data are more sensitive to extracellular pathways and may therefore be useful when edema, fluid redistribution, or low-frequency tissue conduction are of interest. The 50 kHz data provide a familiar clinical reference point and showed the clearest presentation of R normalization with persistent Xc suppression. The 1000 kHz data provide insight into higher-frequency behavior, which may be more influenced by total conductive tissue compartments.

Taken together, these figures suggest that rehabilitation monitoring should not rely on a single frequency or variable. The most informative pattern was the convergence of evidence across R, Xc, PhA, and asymmetry outcomes. This multidimensional interpretation aligns with the intended use of A19FBIVA as a segmental clinical monitoring tool rather than a localized knee assessment or a simple body composition estimate.

A secondary observation was deterioration of the uninjured limb during the early post-injury period. This supports the concept of contralateral neglect, in which reduced global training exposure and unloading negatively affect the limb commonly used as a reference for symmetry-based assessments [13,14,15,49].

## 4. Discussion

### 4.1. Main Findings

The main finding of this longitudinal case report was that segmental bioelectrical recovery did not occur uniformly across impedance-derived variables. R normalized earlier than Xc and PhA, suggesting that fluid- or volume-related segmental changes may precede normalization of capacitance-related bioelectrical properties. This interpretation remains indirect because A19FBIVA measures the entire lower-limb segment rather than localized knee tissue and lacks structural validation of tissue healing [27,28,29,34,36,41].

### 4.2. Why Functional Criteria May Miss Residual Deficits

Functional tests remain essential for RTP decision-making, but they are effort-dependent and can be influenced by compensation, motivation, pain tolerance, movement strategy, and contralateral limb status [1,6,7,8,9,10,16,17,18]. The present case does not provide concurrent evidence that the athlete met standardized functional RTP criteria while remaining bioelectrically deficient. Therefore, we revised the manuscript to avoid claiming that A19FBIVA identified deficits missed by functional testing. Instead, the results suggest that A19FBIVA may provide a complementary physiological signal that should be interpreted alongside functional, clinical, imaging, and sport-specific assessments.

### 4.3. Clinical Meaning of Reactance Lag

Xc reflects capacitive properties of cell membranes and tissue interfaces and may be influenced by cellular density, membrane integrity, tissue composition, hydration, edema, posture, and measurement conditions [28,32,33,34,42,43,44]. Therefore, the persistence of an Xc deficit at T3 is interpreted cautiously as an observational segmental bioelectrical pattern, not as direct evidence of delayed cellular or membrane repair. This may still be clinically relevant in elite alpine skiing, where the lower limbs must withstand high eccentric forces, repeated loading cycles, vibration, and unpredictable environmental demands.

### 4.4. Rethinking Limb Symmetry and Contralateral Comparison

The deterioration observed in the uninjured limb reinforces concerns regarding exclusive reliance on contralateral comparison. When the reference limb is affected by detraining, bilateral reductions in performance or tissue quality can mask the true magnitude of the injured limb deficit [13,14,15,50]. Whenever possible, RTP monitoring should incorporate pre-injury baseline values rather than relying solely on side-to-side comparison.

### 4.5. Toward a Bio-Functional RTP Model

**Figure 7:**
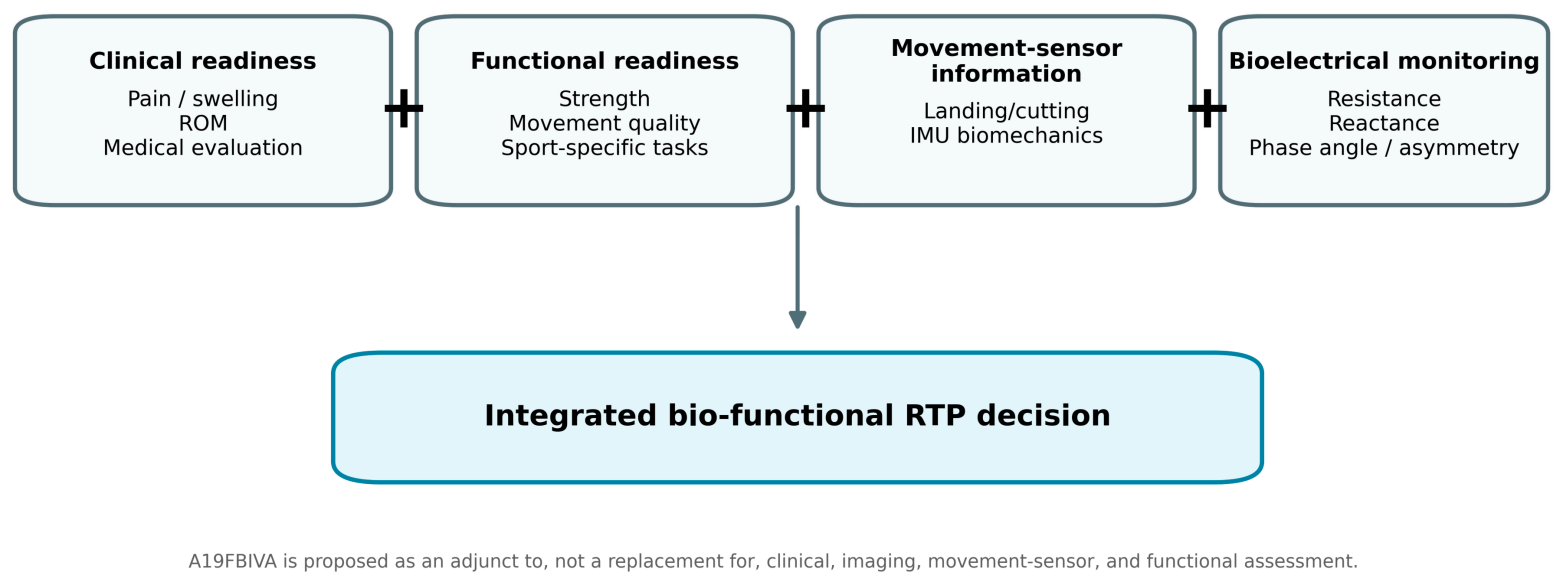
Proposed bio-functional return-to-play (RTP) framework integrating clinical readiness, functional readiness, movement-sensor information, and bioelectrical readiness. Advanced 19-Frequency Bioelectrical Impedance Vector Analysis (A19FBIVA) should be interpreted as a complementary monitoring layer, not a replacement for clinical, imaging, or functional decision-making.

A19FBIVA should not be considered a replacement for functional testing, imaging, clinical examination, or sport-specific assessment. Instead, it may serve as an adjunctive tool that provides resting segmental bioelectrical information during rehabilitation. A bio-functional RTP model may integrate clinical readiness, functional readiness, movement-sensor data, and bioelectrical monitoring, while recognizing that no single measure can independently determine safe sport-specific return. The proposed integrated bio-functional RTP framework is presented in Figure 7.

### 4.6. Exploratory Reference-Line Interpretation

Interpret the −2.5% line only as an exploratory visual reference derived from the present case, not as a validated diagnostic or RTP threshold. The current dataset does not provide device-specific standard error of measurement, minimal detectable change, test–retest reliability, or day-to-day biological variability for segmental PhA. Therefore, we revised the figure and discussion to avoid presenting this value as a clinical cutoff.

Future threshold development should incorporate repeated measurements, technical error, the coefficient of variation, baseline variability, minimal detectable change, sport-specific reference ranges, and prospective outcome data. Until such evidence is available, any small asymmetry should be interpreted cautiously and should not serve as an independent clearance criterion.

### 4.7. Implications for Sensor-Based Rehabilitation Monitoring

The present case illustrates how multifrequency impedance sensing may be integrated into rehabilitation as a repeated resting-monitoring layer. Unlike maximal strength, hop, cutting, or jump-landing tests, A19FBIVA can be performed at rest and may therefore be feasible during phases when high-load testing is contraindicated or undesirable. It should not be interpreted as a stand-alone readiness test but as a source of complementary segmental physiological information.

Within the broader sensor-based rehabilitation framework, A19FBIVA may complement wearable technologies such as IMUs. IMUs quantify lower-limb biomechanics during dynamic tasks, including landing and cutting, whereas A19FBIVA provides resting segmental bioelectrical information [48]. Combining these approaches may help clinicians distinguish movement capability, clinical status, and physiological recovery, provided each technology is interpreted within its own validity and reliability limits.

Implementation requires strict standardization. Measurement timing, pre-test conditions, device type, electrode configuration, posture, weight-bearing, reporting frequency, and asymmetry calculation must be consistent. Without this standardization, small longitudinal differences may reflect protocol noise rather than true physiological change. Future research should therefore report raw values, repeated-measures reliability, percentage changes, device details, and measurement conditions, rather than only derived summaries.

## 5. Limitations and Future Directions

Although the single-case design is suitable for hypothesis generation, several methodological issues require attention before A19FBIVA can serve as a robust RTP decision-support tool. Future studies should include larger cohorts, diverse injury types, both sexes, multiple sports, and standardized follow-up beyond RTP. Ideally, A19FBIVA should be collected alongside MRI or ultrasound, isokinetic strength testing, jump and hop testing, patient-reported outcomes, wearable sensor-based movement assessments, and subsequent reinjury surveillance.

A key priority is to determine whether persistent Xc or PhA deficits predict delayed performance recovery, incomplete tissue remodeling, or increased reinjury risk. It is also necessary to establish typical day-to-day variability, technical error, coefficient of variation, standard error of measurement, and smallest detectable change values for elite athletes. Without these reliability data, exploratory reference values such as −2.5% should not be used as independent clearance criteria.

This study has several limitations. First, the findings are based on a single athlete and should therefore be interpreted as hypothesis-generating rather than definitive. Second, the Seca mBCA 515 measures impedance of the entire lower-limb segment rather than localized knee tissue; consequently, the observed changes may reflect combined effects of muscle mass, hydration, edema, connective tissue, and other tissues throughout the leg. Third, no concurrent imaging, isokinetic strength testing, standardized functional RTP tests, or patient-reported outcome measures were available at each time point, so the relationship between bioelectrical recovery and functional performance could not be directly quantified. Fourth, repeated measurement sweeps were not available to calculate technical error, coefficient of variation, SEM, MDC, or test–retest reliability. Fifth, hydration was standardized only pragmatically, and detailed food/fluid intake, body mass changes, medication use, environmental conditions, recent training load, and menstrual-cycle phase were not fully recorded. Finally, the results are specific to the measurement protocol and device used, and transferability to other bioimpedance systems requires further investigation [1,8,10,51].

## 6. Conclusions

This longitudinal case report suggests that A19FBIVA may provide clinically relevant segmental bioelectrical information during post-injury rehabilitation monitoring in elite sport. In an elite alpine skier recovering from a complex knee injury, R normalized earlier than Xc and PhA, indicating an observed dissociation between conductive and capacitance-related bioelectrical properties of the injured lower-limb segment. These findings should not be interpreted as direct evidence of localized knee-tissue healing or as proof of RTP readiness. Instead, the observed reactance lag and deterioration in the contralateral limb highlight potential limitations of relying exclusively on symmetry-based RTP criteria. A19FBIVA may complement clinical examination, imaging, functional testing, and movement-sensor assessment by adding a layer of resting physiological monitoring to rehabilitation decision-making. Further research is required to validate reliability, thresholds, and predictive value for reinjury risk and performance restoration.

For the Journal of Clinical Medicine, the broader clinical contribution of this report is demonstrating that multifrequency impedance monitoring may support individualized rehabilitation monitoring after severe sports injury when interpreted cautiously and in conjunction with other clinical information. The next step is not only to collect larger datasets but also to determine which sensor-derived variables meaningfully change clinical decisions.

## Figures and Tables

**Table 1:** Participant and injury characteristics.

Variable	Description
Sport	Alpine skiing
Competitive level	Elite/Slovenian national level
Sex	Female
Injured limb	Right lower limb
Injury location	Right knee
Injury type	Tibial fracture with associated ligamentous, tendinous, and muscular strain; detailed fracture classification and full management documentation were not available
Immobilization	Approximately one-month rest period with non-walking knee brace
Rehabilitation	Medically supervised progressive rehabilitation
Primary monitoring tool	A19FBIVA using Seca mBCA 515

**Table 2:** Advanced 19-Frequency Bioelectrical Impedance Vector Analysis (A19FBIVA) measurement protocol.

Component	Description
Device	Seca mBCA 515
Measurement type	Segmental multifrequency bioelectrical impedance
Electrode system	Eight-electrode configuration
Frequencies	1, 1.5, 2, 3, 5, 7.5, 10, 15, 20, 30, 50, 75, 100, 150, 200, 300, 500, 750, and 1000 kHz
Body segments	Left leg, right leg, left arm, right arm, trunk, left body side, right body side, whole body
Position	Standing, barefoot, hands gripping electrodes; symmetrical posture and full electrode contact visually checked; no quantitative load-distribution data available
Pre-test control	Similar time of day, resting state, no intense exercise < 12 h, normal hydration, bladder voided; food/fluid intake, medication, environment, detailed training load, body mass, and menstrual-cycle phase not fully recorded
Main variables	Resistance, reactance, phase angle, inter-limb asymmetry

**Table 3:** Main longitudinal bioelectrical outcomes in the injured right leg at 50 kHz.

Time Point	Timing	RL R Change (%)	RL Xc Change (%)	Interpretation
T1	26 days post-injury	+11.67	−3.05	Disuse-related changes after immobilization
T2	64 days post-injury	+2.10	−12.05	Resistance improving, reactance lagging
T3	135 days post-injury	−2.61	−11.36	Resistance normalized/improved, reactance deficit persists

RL—right leg; R—resistance; Xc—reactance.

**Table 4:** Phase angle outcomes at 50 kHz.

Time Point	RL vs. LL PhA Asymmetry (%)	RL PhA Deficit vs. Baseline (%)	Interpretation
T0	+1.14	0.00	High symmetry/pre-injury reference
T1	−17.38	−18.93	Severe membrane-related deficit after immobilization
T2	−7.54	−12.68	Partial recovery
T3	−3.40	−9.51	Residual segmental PhA deficit at final late-stage rehabilitation follow-up

RL—right leg; LL—left leg; PhA—phase angle.

## Data Availability

The data presented in this study are available on request from the corresponding author due to privacy and ethical restrictions associated with a longitudinal single-case report involving an elite athlete. Please direct requests for access to the corresponding author, and we will consider them in accordance with the participant’s consent and applicable institutional data protection requirements. The first author, Stanislav Štuhec, may also be contacted regarding access to the raw data at stanislav.stuhec@fsp.uni-lj.si.

## Supplementary Materials

The following supporting information can be downloaded at: https://www.mdpi.com/article/10.3390/jcm15187231/s1, Figure S1: Resistance and reactance at 1 kHz between four consecutive measurements; Figure S2: Resistance and reactance at 1000 kHz between four consecutive measurements.
