# Supplementary material for: Bioelectrical Recovery After Knee Injury in an Elite Alpine Skier: A Longitudinal 19-Frequency Bioelectrical Impedance Vector Analysis Case Report
Source: J Clin Med. 2026 Sep 17;15(18):7231. doi: 10.3390/jcm15187231 (PMC13607350; doi:10.3390/jcm15187231)
Supplement: Supplementary file 1 [file jcm-15-07231-s001.zip › jcm-4527059-supplementary.pdf]

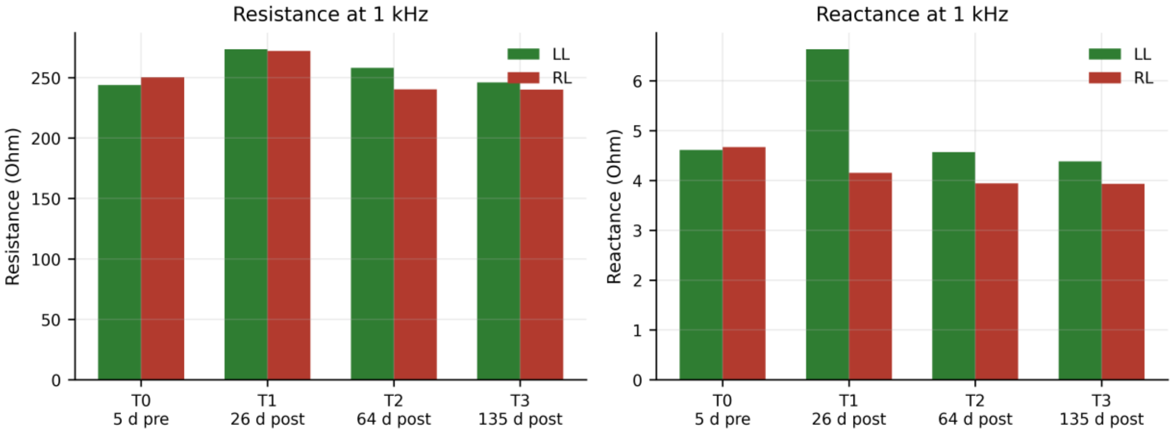

Figure S1. Resistance and reactance at 1 kHz between four consecutive measurements.

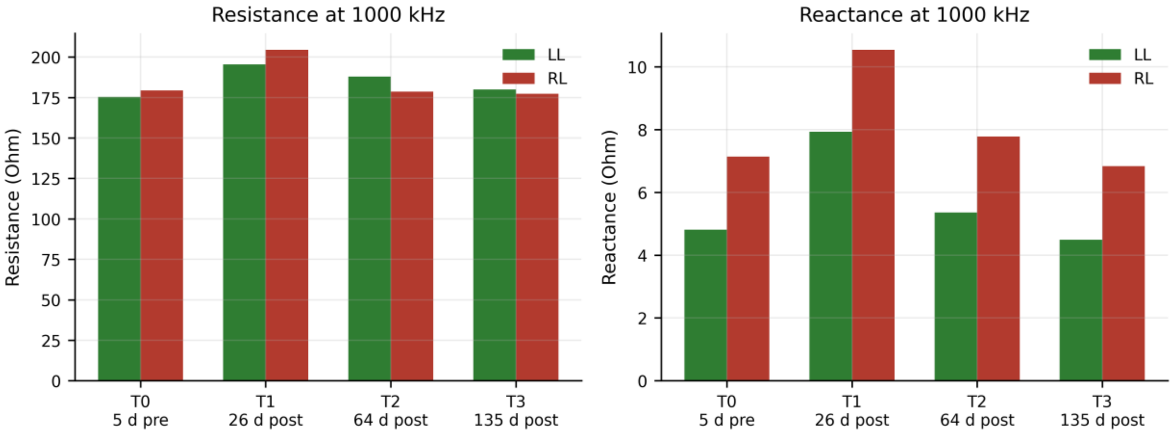

Figure S2. Resistance and reactance at 1000 kHz between four consecutive measurements.
